# Polyp bailout in *Pocillopora damicornis* following thermal stress

**DOI:** 10.12688/f1000research.11522.2

**Published:** 2017-08-10

**Authors:** Alexander J Fordyce, Emma F Camp, Tracy D Ainsworth

**Affiliations:** 1 ARC Centre of Excellence for Coral Reef Studies, James Cook University, Queensland, Australia; 2 Climate Change Cluster, University of Technology Sydney, New South Wales, Australia

**Keywords:** polyp bailout, thermal stress, coral bleaching, Pocillopora damicornis

## Abstract

Polyp bailout is an established but understudied coral stress response that involves the detachment of individual polyps from the colonial form as a means of escaping unfavourable conditions. This may influence both the mortality and asexual recruitment of coral genotypes across a range of species. It has been observed in response to numerous stressors including high salinity and low pH. Polyp expulsion in association with thermal stress has once been described in a geographically restricted, temperate species. We therefore cannot reliably apply this observation to tropical coral reefs around the world, which are increasingly under threat from thermal stress events. We present the first qualitative observation of polyp bailout following acute temperature shock  in a near-natural mesocosm experiment. Detached polyps show similar characteristics to those described in previous studies, including the retention of endosymbiotic zooxanthellae and the ability to disperse across short distances. This finding strongly suggests that polyp bailout occurs in tropical coral reef environments and warrants further detailed research into the implication of this response in terms of individual survival, rapid migration into cooler micro-habitats and local recruitment within the reef environment and its coral community.

## Introduction

Coral reefs around the world are facing increasingly frequent acute thermal stress events ( Ainsworth *et al.*, 2016; Hughes *et al.*, 2017). As such there has been a corresponding increase in research into how corals respond to high temperatures, how these responses vary within individuals and communities, and how variation influences the resilience, recovery and structure of coral communities. Understanding this variation helps predict patterns of bleaching-induced mortality and reef-wide degradation. As the possibility of frequent, severe bleaching events increases ( van Hooidonk *et al.*, 2016), it is important to understand the drivers of variability in order to improve management and target restoration efforts. Polyp bailout is a possible source of variation that may influence the survival of individual genotypes and recruitment at local scales.

Polyp bailout has been observed in at least six species of tropical scleractinian coral ( Serrano *et al.*, 2017) and involves the withdrawal of individual polyps from the coenosarc followed by their detachment from the skeleton ( Sammarco, 1982). The detachment of individual polyps from a parent coral colony has previously been recorded in response to poor water quality ( Sammarco, 1982; Serrano *et al.*, 2017), large changes in pH ( Kvitt *et al.*, 2015), changes in salinity ( Shapiro *et al.*, 2016) and following competition from macroalgae ( Sin *et al.*, 2012). As polyps often retain their endosymbiotic dinoflagellates (zooxanthellae) and are able to re-settle, polyp bailout is thought to be a generalised escape response from detrimental conditions ( Kvitt *et al.*, 2015; Sammarco, 1982). It may therefore constitute rapid migration away from local sources of mortality.

Despite increasing temperatures being arguably the most significant threat to tropical coral reefs ( Hughes *et al.*, 2017), no tropical species have been observed to respond to thermal stress in this way. Kružić (2007) briefly described polyp expulsion in the temperate species *Cladocora caespitosa* following severe thermal stress. However, given the relatively small range of this species, small sample size (n=2) used and lack of information regarding experimental set up, the results are not necessarily applicable to tropical coral reefs around the world. Here we present the first qualitative observation of polyp bailout following thermal stress in *Pocillopora damicornis*, a common reef-forming tropical species ( Hoeksema *et al.*, 2014), during an *ex situ* mesocosm study.

## Methods

Colonies of *P. damicornis* (n=32) were collected from the Heron Island reef flat in January 2017, from a maximum depth of two metres. Each colony was quartered to give a total of 128 fragments. They were housed in four 500 litre aquaria as part of an outdoors, semi-closed system supplied by a continuous flow of unfiltered seawater from the reef flat. During the week preceding simulated thermal stress, all fragments were acclimated to the aquaria and subjected to ambient conditions ( *ca*. 7.980 – 8.020 pH; conductivity of 53 – 54 μS/m; temperature of 26 – 30°C; and PAR of 0 – 3875 K). Following this, temperature was gradually increased in two mesocosms (n=64) on top of natural variation for six days up to a peak daytime temperature of 34°C to simulate a severe bleaching event (as previously reported by Ainsworth *et al.*, 2016). Two control mesocosms continued to be exposed to ambient conditions, differing from treatments in temperature only. Fragments were monitored throughout the day and when polyps were observed to bail out, they were collected using a wide-ended pipette and examined under an Olympus SZX16 stereomicroscope.

## Results

**Figure 1.**
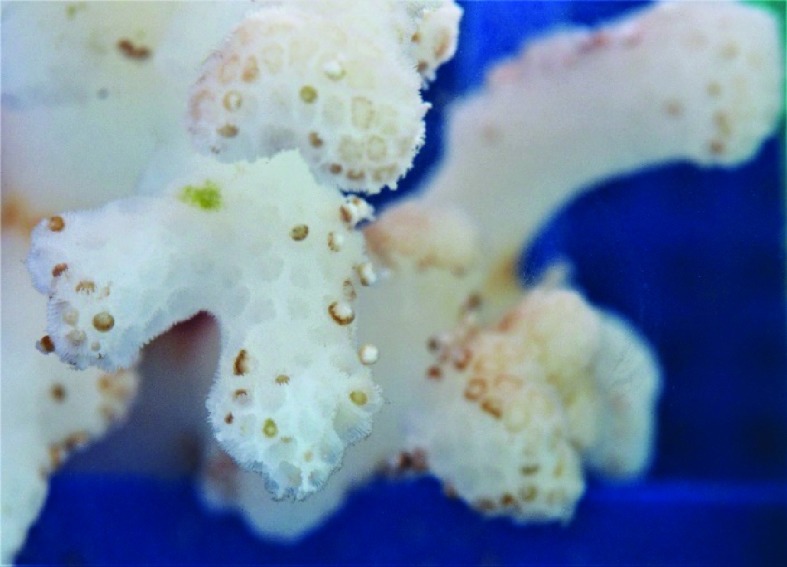
Photographs of polyps dropping off the skeleton of *Pocillopora damicornis.* Macrophotograph of polyps, having withdrawn from the connective coenosarc, dropping off the skeleton of the fragments of *Pocillopora damicornis*. Photograph taken with an Olympus Stylus Tough TG-4.

On the fifth day of the simulated bleaching event, polyps were observed to begin bailing out at approximately 09:30 ( Figure 1; Dataset 1, Fordyce *et al.*, 2017). At this time, peak daytime temperature was 33°C, equivalent to 13 degree heating days (DHDs). DHDs are a measure of accumulated heat stress that is calculated as the cumulative temperature increases above the mean monthly maximum across a period of time. The more common measure, Degree Heating Weeks (DHWs), is used in the prediction and assessment of mass bleaching events (e.g. Ainsworth *et al.*, 2016). By the end of day six, at peak temperature of 34°C, reflecting 18 degree heating days, all polyps had detached ( Dataset 1, Fordyce *et al.*, 2017). At the end of the bailout period, polyps began to detach in sheets rather than as individuals. This suggests that thermal stress was too severe to allow successful withdrawal of all polyps from the coenosarc. In contrast, fragments in the control mesocosms showed no signs of bleaching or polyp bailout ( Supplementary Material 1).

**Figure 2.**
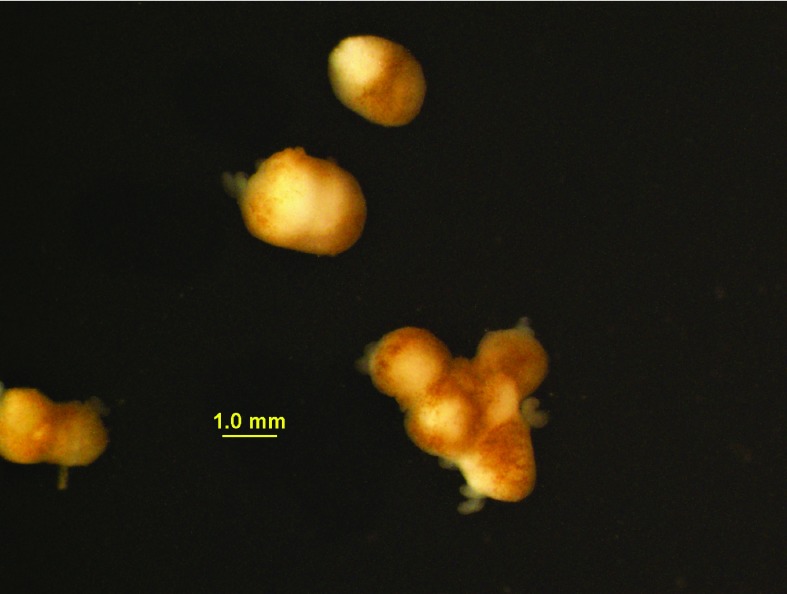
Single polyps and clusters of polyps with zooxanthellae and extended filaments. Micrograph of single polyps and clusters of polyps placed in a glass petri dish, taken using an Olympus SZX16 stereomicroscope with a computer-linked 2.0x objective lens. Total magnification is 17.0x. Small brown dots in the polyp tissue are endosymbiotic zooxanthellae. Coiled filaments are thought to be mesenteries which may aid in resettlement, through adhesion, or feeding.

Bailed polyps were slightly negatively buoyant, sinking slowly, but could easily be re-suspended with mild disturbance. The detached, individual polyps retained their zooxanthellae and many were observed to extend what are likely to be mesenterial filaments (as described in Richmond, 1985; Figure 2; Supplementary Material 2). Clusters of detached polyps were also observed, however these lacked calcified tissue and so did not resemble the larval clusters described by Richmond (1985) ( Figure 2).

Table of qualitative observations of polyp bailout in control and heat-treated mesocosms Indicated is the peak daytime temperature (± 0.5°C) of the four treated mesocosms, the accumulated heat stress corals are exposed to and any observations during the four day bleaching period, including at the beginning and end of polyp bailout. Click here for additional data file. Copyright: © 2017 Fordyce AJ et al. 2017 Data associated with the article are available under the terms of the Creative Commons Zero "No rights reserved" data waiver (CC0 1.0 Public domain dedication).

## Implications and future research

In past observations of polyp bailout, corals have largely been subjected to extreme aquarium conditions such as high salinity (up to 54 parts per thousand; Shapiro *et al.*, 2016), low pH (7.2; Kvitt *et al.*, 2015) or little to no water replacement resulting in anoxic and low nutrient conditions ( Serrano *et al.*, 2017; Sin *et al.*, 2012). Kružić (2007) on the other hand examined a temperate Mediterranean species. This makes it difficult to apply these results to the context of natural tropical reefs and elucidate the possible role of this response during environmental stress. The present observation was in aquaria with near-natural conditions, using an important reef coral species common across the Indo-Pacific. The peak daily temperature of 34°C was sustained over several days, reflecting severe thermal stress. However, accumulated heat stress amounted to between two and three degree heating weeks (14–21 degree heating days). This is becoming widely reported during bleaching events on the Great Barrier Reef ( Ainsworth *et al.*, 2016; Hughes *et al.*, 2017). Therefore, this observation suggests that polyp bailout may be widespread in natural reef environments, in response to currently observed temperature increases. For coral species that utilise this response, the present record has implications for coral recruitment and recovery on local scales, and also suggests that these processes can occur independent of sexual reproduction and the impact of thermal stress on reproductive potential.

Resettlement of detached polyps was not observed, however they appear to be viable and may be able to settle near the parent colony or be dispersed short distances within a reef environment. Sammarco (1982) previously described low (< 5%) settlement and survival rates of detached polyps on settlement tiles contained in jars but survival of individual polyps within a simulated or natural reef environment has yet to be investigated, particularly how it is affected by reef degradation. If polyps were to ‘escape’ into cooler conditions with higher food availability, it may boost their survival and settlement success rates beyond the 5% observed by Sammarco. Indeed, Shapiro *et al.* (2016) reported far higher rates of resettlement (≥ 50%) when settling detached polyps under more favourable conditions, indicating that the low settlement observed by Sammarco can be partly explained by the environment in which resettlement was tested. In the current study, we noted that each fragment detached a large population of individual polyps (>50 per fragment), showing that a survival of 50% has the potential to allow for some immediate and significant re-seeding of the local reef habitat. Furthermore, an obvious source of nearby refugia are neighbouring reef slope and mesophotic reef environments ( Bridge *et al.*, 2013; Smith *et al.*, 2014), down to which these negatively buoyant polyps may slowly sink. This is particularly relevant given that Kramarsky-Winter *et al.* (1997) only observed polyp expulsion, associated with asexual reproduction, in waters ≤ 7 m; in the present study, colonies were collected from waters ≤ 2 m. However, light attenuation may limit viable settlement depths as individual polyps would need to rapidly acclimate to lower light conditions in order to settle and successfully begin asexual division.

Clearly, extensive future research is needed to explore the survival of individual polyps in both simulated refugia and within the reef habitat following thermal stress events. This will lead to greater understanding of the ecology and wider implications of this stress response and its potential role in coral recruitment and reef recovery following bleaching events. We additionally lack *in situ* observations of this phenomenon. Time-intensive ecological surveying during a predicted bleaching event is needed to reveal whether this is a widespread response to thermal stress. Subsequent use of mass mark and recapture of coral polyps, tagged with stable heavy isotopes, would then allow the tracking of the fate of coral polyps following bailout. Despite being a well-established response to stress, little research has focused on how polyp bailout may influence the survival and recovery of local coral populations.

## Data availability

The data referenced by this article are under copyright with the following copyright statement: Copyright: © 2017 Fordyce AJ et al.

Data associated with the article are available under the terms of the Creative Commons Zero "No rights reserved" data waiver (CC0 1.0 Public domain dedication). 

**Dataset 1. Table of qualitative observations of polyp bailout in control and heat-treated mesocosms.** Indicated is the peak daytime temperature (± 0.5°C) of the four treated mesocosms, the accumulated heat stress corals are exposed to and any observations during the four day bleaching period, including at the beginning and end of polyp bailout.

DOI, 10.5256/f1000research.11522.d161213 ( Fordyce *et al.*, 2017)

## Supplementary Material

**Supplementary Material 1. Comparative photographs of control and bleached fragments to show bleaching.** Photographs were taken using an Olympus Stylus Tough TG-4 camera. A) Photograph of control fragment taken on 31/01/17; B) Photograph of bleached fragment, with visibly withdrawn individual polyps still attached to the skeleton, taken on 30/01/17.

.

**Supplementary Material 2. Micrograph of a single polyp with an extended filament.** Micrograph of a single polyp taken using the same techniques and equipment used for Figure 2, more clearly showing a coiled, extended filament. Based on the findings of Richmond (1985), this is thought to be a mesentery.

## References

[ref-1] Ainsworth TD Heron SF Ortiz JC: Climate change disables coral bleaching protection on the Great Barrier Reef. *Science.* 2016;352(6283):338–342. 10.1126/science.aac7125 27081069

[ref-2] Bridge TC Hoey AS Campbell SJ: Depth-dependent mortality of reef corals following a severe bleaching event: implications for thermal refuges and population recovery [version 3; referees: 2 approved, 1 approved with reservations]. *F1000Res.* 2013;2:187. 10.12688/f1000research.2-187.v3 24627789 PMC3938179

[ref-3] Fordyce AJ Camp EF Ainsworth TD: Dataset 1 in: Polyp bailout in *Pocillopora damicornis* following thermal stress. *F1000Research.* 2017 Data Source 10.12688/f1000research.11522.1 PMC5580424 28928944

[ref-14] Hoeksema BW Rogers A Quibilan MC: *Pocillopora damicornis.* The IUCN Red List of Threatened Species 2014.2014;e.T133222A54216898 10.2305/IUCN.UK.2014-1.RLTS.T133222A54216898.en

[ref-5] Hughes TP Kerry JT Álvarez-Noriega M: Global warming and recurrent mass bleaching of corals. *Nature.* 2017;543(7645):373–377. 10.1038/nature21707 28300113

[ref-15] Kramarsky-Winter E Fine M Loya Y: Coral polyp expulsion. *Nature.* 1997;387:137 10.1038/387137a0

[ref-16] Kružić P: Polyp expulsion of the coral *Cladocora caespitosa* (Anthozoa, Scleractinia) in extreme sea temperature conditions. *Natura Croatica.* 2007;16(3):211–214. Reference Source

[ref-6] Kvitt H Kramarsky-Winter E Maor-Landaw K: Breakdown of coral colonial form under reduced pH conditions is initiated in polyps and mediated through apoptosis. *Proc Natl Acad Sci U S A.* 2015;112(7):2082–2086. 10.1073/pnas.1419621112 25646434 PMC4343167

[ref-7] Richmond RH: Reversible metamorphosis in coral planulae larvae. *Mar Ecol Prog Ser.* 1985;22:181–185. 10.3354/meps022181

[ref-8] Sammarco PW: Polyp bail-out: an escape response to environmental stress and a new means of reproduction in corals. *Mar Ecol Prog Ser.* 1982;10:57–65. 10.3354/meps010057

[ref-9] Serrano E Coma R Inostroza K: Polyp bail-out by the coral *Astroides calycularis* (Scleractinia, Dendrophylliidae). *Mar Biodiv.*Published online.2017;1–5. 10.1007/s12526-017-0647-x

[ref-10] Shapiro OH Kramarsky-Winter E Gavish AR: A coral-on-a-chip microfluidic platform enabling live-imaging microscopy of reef-building corals. *Nat Commun.* 2016;7: 10860. 10.1038/ncomms10860 26940983 PMC4785229

[ref-11] Sin LC Walford J Goh BP: The effect of benthic macroalgae on coral settlement. *Contrib Mar Sci.* 2012;2012:89–93. 10.13140/RG.2.1.5133.6801

[ref-12] Smith TB Glynn PW Maté JL: A depth refugium from catastrophic coral bleaching prevents regional extinction. *Ecology.* 2014;95(6):1663–1673. 10.1890/13-0468.1 25039230

[ref-13] van Hooidonk R Maynard J Tamelander J: Local-scale projections of coral reef futures and implications of the Paris Agreement. *Sci Rep.* 2016;6: 39666. 10.1038/srep39666 28000782 PMC5175274

