# Supplementary material for: Polyp bailout in Pocillopora damicornis following thermal stress
Source: F1000Res. 2017 Aug 10;6:687. Originally published 2017 May 17. [Version 2] doi: 10.12688/f1000research.11522.2 (PMC5580424; doi:10.12688/f1000research.11522.2)
Supplement: Table of qualitative observations of polyp bailout in control and heat-treated mesocosms — Indicated is the peak daytime temperature (± 0.5°C) of the four treated mesocosms, the accumulated heat stress corals are exposed to and any observations during the four day bleaching period, including at the beginning and end of polyp bailout. [file f1000research-6-13393-s0000.tgz › 9b9a5aba-166f-4fb6-9fe8-71864ddb186c_Dataset_1.pdf]

Dataset 1. Table of qualitative observations of polyp bailout in control and heat-treated mesocosms. Indicated is the peak daytime temperature (± 0.5°C) of the four treated mesocosms, the accumulated heat stress corals are exposed to and any observations during the four day bleaching period, including at the beginning and end of polyp bailout.

			**Accumulated Heat Stress**		
**Date**	Tank	Peak daytime Temperature (°C) (± 0.5)	Degree Heating Days	Degree Heating Weeks	Notes
**19/01/17**	Heat 1	29	0	0	Acclimation period
**19/01/17**	Heat 2	29	0	0	
**19/01/17**	Control 1	29	0	0	
**19/01/17**	Control 2	29	0	0	
**20/01/17**	Heat 1	29	0	0	
**20/01/17**	Heat 2	29	0	0	
**20/01/17**	Control 1	29	0	0	
**20/01/17**	Control 2	29	0	0	
**21/01/17**	Heat 1	29	0	0	
**21/01/17**	Heat 2	29	0	0	
**21/01/17**	Control 1	29	0	0	
**21/01/17**	Control 2	29	0	0	
**22/01/17**	Heat 1	29	0	0	
**22/01/17**	Heat 2	29	0	0	
**22/01/17**	Control 1	29	0	0	
**22/01/17**	Control 2	29	0	0	
**23/01/17**	Heat 1	29	0	0	
**23/01/17**	Heat 2	29	0	0	
**23/01/17**	Control 1	29	0	0	
**23/01/17**	Control 2	29	0	0	
**24/01/17**	Heat 1	29	0	0	
**24/01/17**	Heat 2	29	0	0	
**24/01/17**	Control 1	29	0	0	
**24/01/17**	Control 2	29	0	0	

**25/01/17**	Heat 1	29	0	0	
**25/01/17**	Heat 2	29	0	0	
**25/01/17**	Control 1	29	0	0	
**25/01/17**	Control 2	29	0	0	
**26/01/17**	Heat 1	30	1	0.1	Temperature increase begins
**26/01/17**	Heat 2	30	1	0.1	
**26/01/17**	Control 1	29	0	0	
**26/01/17**	Control 2	29	0	0	
**27/01/17**	Heat 1	31	3	0.4	
**27/01/17**	Heat 2	31	3	0.4	
**27/01/17**	Control 1	29	0	0	
**27/01/17**	Control 2	29	0	0	
**28/01/17**	Heat 1	32	6	0.9	Paling first observed in coral tissue, not bailout or loss of tissue
**28/01/17**	Heat 2	32	6	0.9	
**28/01/17**	Control 1	29	0	0	
**28/01/17**	Control 2	29	0	0	
**29/01/17**	Heat 1	33	9	1.3	Mild paling almost all coral fragments
**29/01/17**	Heat 2	33	9	1.3	
**29/01/17**	Control 1	29	0	0	
**29/01/17**	Control 2	29	0	0	
**30/01/17**	Heat 1	33	13	1.9	Polyp bailout first observed in late morning (~09:30 am) and continued throughout the day. Appeared to slow down in the early evening, approximately 6 pm. Majority of fragments were ~50% bleached by the end of the day.
**30/01/17**	Heat 2	33	13	1.9	
**30/01/17**	Control 1	29	0	0	
**30/01/17**	Control 2	29	0	0	
**31/01/17**	Heat 1	34	18	2.6	

**31/01/17**	Heat 2	34	18	2.6	All corals fully bleached/bailed by 4 pm. In the last few hours, thermal stress appeared to be too severe to allow all polyps to withdraw and detach as individuals; large sheets of 10+ polyps began to fall off.
**31/01/17**	Control 1	29	0	0	
**31/01/17**	Control 2	29	0	0	
