# Supplementary figures and images for: Polyp bailout in Pocillopora damicornis following thermal stress
Source: F1000Res. 2017 Aug 10;6:687. Originally published 2017 May 17. [Version 2] doi: 10.12688/f1000research.11522.2 (PMC5580424; doi:10.12688/f1000research.11522.2)

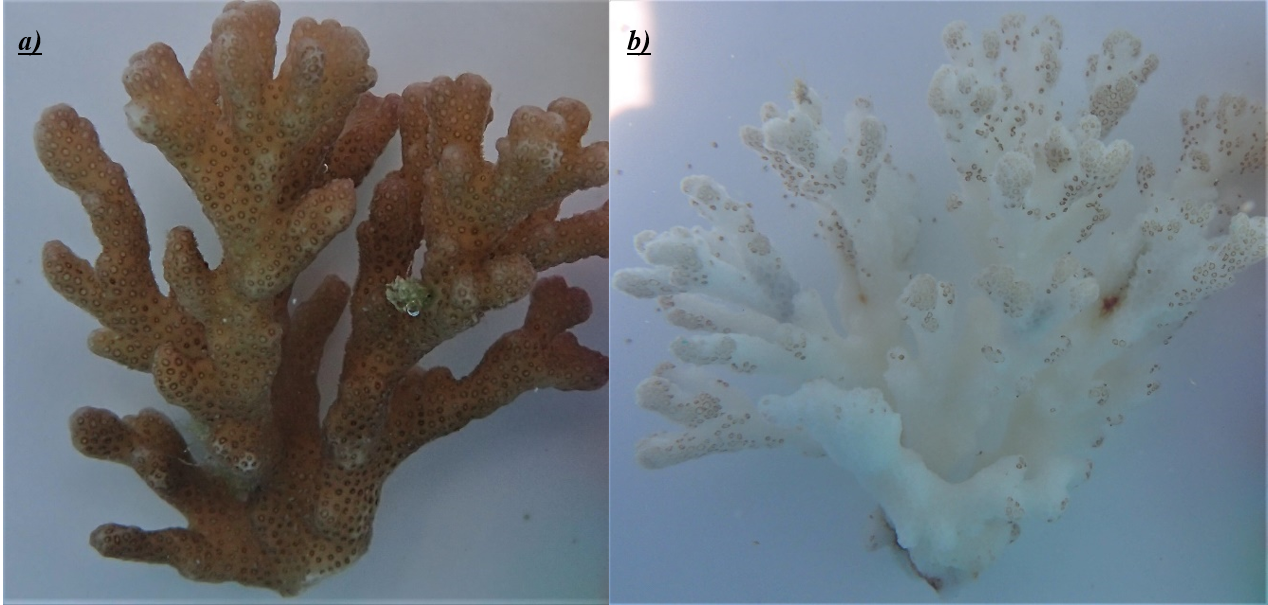

Supplement: Supplementary file 2 [file f1000research-6-13393-s0001.tgz › 2b0eb6e3-8c53-4e51-b84b-fd04dfbf706c.tif]

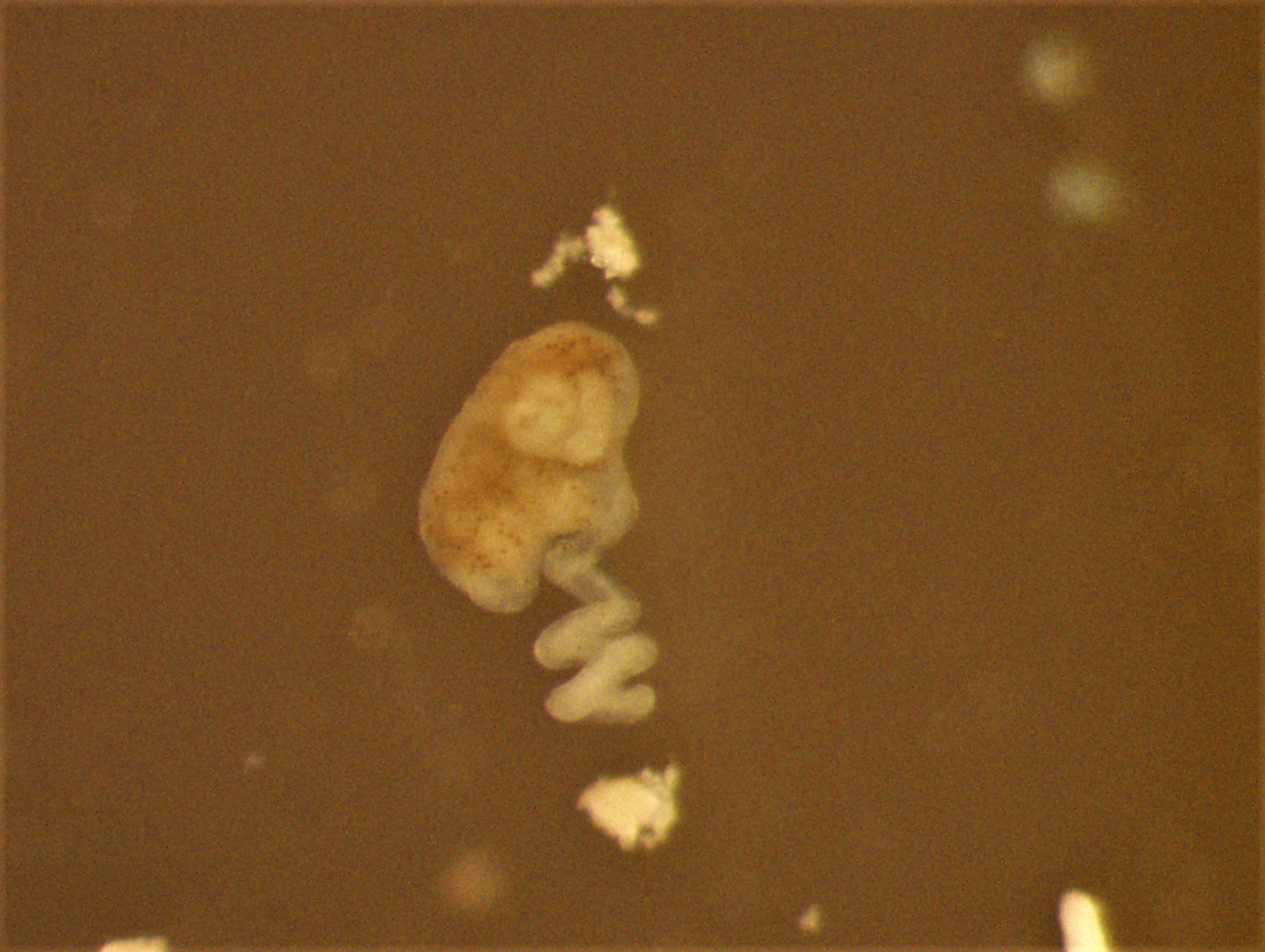

Supplement: Supplementary file 3 [file f1000research-6-13393-s0002.tgz › f13f8cd8-0ae3-4403-a8ba-68c2865a5d1b.tif]
