# Supplementary material for: Polyp bailout in  Pocillopora damicornis following thermal stress
Source: F1000Res. 2017 Aug 10;6:687. Originally published 2017 May 17. [Version 2] doi: 10.12688/f1000research.11522.2 (PMC5580424; doi:10.12688/f1000research.11522.2)
Supplement: Peer review history

# Peer review: Referee response for version 2

**DOI:** 10.5256/f1000research.13393.r24886

**Published:** 2017-08-29

**Authors:** Dan Tchernov, Hagit Kvitt

**Affiliations:** 1 School of Marine Sciences, University of Haifa, Haifa, Israel; 2 School of Marine Sciences, Haifa University, Haifa, Israel

It ​is properly revised, and therefor approved for publication.​

We have read this submission. We believe that we have an appropriate level of expertise to confirm that it is of an acceptable scientific standard.

# Peer review: Referee response for version 1

**DOI:** 10.5256/f1000research.12446.r22841

**Published:** 2017-07-24

**Authors:** Dan Tchernov, Hagit Kvitt

**Affiliations:** 1 School of Marine Sciences, University of Haifa, Haifa, Israel; 2 School of Marine Sciences, Haifa University, Haifa, Israel

General remarks: 

1. In the Abstract the authors claim: ”However, polyp bailout has not previously been described in association with thermal stress and the coral bleaching response…”
  a. This is completely inaccurate-polyp bailout or expulsion in response to thermal stress has been reported in 2007 by Kruzic P.
  b. Moreover, the claim that bleaching is also involved: “in association with thermal stress and the coral **bleaching response**” is misleading - there is not a single experimental proof or measurement of bleaching in the current report, merely some remarks on “Paling observed in coral tissue” and “All corals fully bleached" (Dataset 1.). Moreover, the polyps are clearly detaching with the zooxanthellae, (Figure 2: “Small brown dots in the polyp tissue are endosymbiotic zooxanthellae”). So where is the bleaching? In the coenosarc? If so - experimental evidence should be provided. Or the sentence: “..the coral **bleaching response**” should have been omitted.
2. In the Results, the use of the term “heating days” is unclear, and should have been explained (“At this time, peak daytime temperature was 33°C, which is the **equivalent of 13 degree heating days, a measure of accumulated heat stress used in the prediction of mass bleaching events** “).
3. Figure 2 is unclear, especially the presumed “coiled filaments are adhesive mesenterial filaments, presumed to aid in rapid settlement”. I would expect, under current technologies available, for a better image to convince that indeed these are “adhesive mesenterial filaments”.

 

 The literature is not correctly cited.

1. In the Abstract, the paper by Goreau & Goreau (1959) does not clearly describe polyp bailout or expulsion. It deals with calcification all over, and it mentions merely in the Discussion, as unpublished results, that polyps can detach. This citation should have been be omitted from the Abstract and put elsewhere.
2. The paper of Kramarsky-Winter *et al.* 1997 on polyp expulsion is not cited.
3. The paper of Kruzic P. 2007 on polyp expulsion under thermal stress, which is the most relevant paper to this report, is not cited.

We have read this submission. We believe that we have an appropriate level of expertise to confirm that it is of an acceptable scientific standard, however we have significant reservations, as outlined above.

# Peer review: Referee response for version 1

**DOI:** 10.5256/f1000research.12446.r23072

**Published:** 2017-06-19

**Authors:** Sylvain Agostini

**Affiliations:** 1 Shimoda Marine Research Center, University of Tsukuba, Shizuoka, Japan

This paper reports on the qualitative of polyp bailout for the coral species Pocillopora damicornis under heat stress in mesocosms.

 The study remains qualitative and the authors are clear on this point.

 The only remark I would have is in the discussion. The sentence "Detached polyps appear to be viable, able to settle near the parent colony and capable of being dispersed short distances in a reef environment." sounds like that resettlement of the bailed polyp were observed. However it was not observed and remain a speculation. I would suggest to rephrase it to something like that:

 "While resettlement of the bailed out polyps could not be observed, the polyps appear to be viable, and could be able to settle near the parent colony and to be dispersed accross short distances in a reef environment."

I have read this submission. I believe that I have an appropriate level of expertise to confirm that it is of an acceptable scientific standard.
